# Verbascoside attenuates experimental varicocele-induced damage to testes and sperm levels through up-regulation of the hypothalamus-pituitary-gonadal (HPG) axis

**DOI:** 10.1080/13880209.2021.1933085

**Published:** 2021-06-19

**Authors:** Letian Han, Shan Xiang, Baohai Rong, Yanchen Liang, Shengtian Zhao

**Affiliations:** a The First Clinical School, Shandong University of Traditional Chinese Medicine, Jinan, China; b Reproductive and Genetic Center of Integrated Traditional and Western Medicine, The Affiliated Hospital of Shandong University of Traditional Chinese Medicine, Jinan, China; c Department of General Surgery, The Affiliated Hospital of Shandong University of Traditional Chinese Medicine, Jinan, China; d Department of Orthopedics, The Affiliated Hospital of Shandong University of Traditional Chinese Medicine, Jinan, China; e Department of Urology, Affiliated Hospital of Shandong University of Traditional Chinese Medicine, Jinan, China

**Keywords:** Antioxidant, gonadotropin-releasing hormone, sperm count, luteinizing hormone, infertility

## Abstract

**Context:**

Verbascoside (VB), which is found in many medicinal plant families, exhibits biological activities in various diseases. However, its effects on varicocele (VCL)-induced damage remain unknown.

**Objective:**

To investigate the effects and mechanism of VB on experimental rats with varicocele (VCL)-induced damage.

**Materials and methods:**

Sixty sexually mature male Sprague-Dawley (SD) rats were divided into six groups (*n* = 10): control, control-sham, VCL-vehicle (normal saline), and VCL + VB groups (50, 100, and 200 mg/kg/day, intraperitoneally). After 4 weeks of VB treatment, all animals were sacrificed, and the body and testicular weight, sperm quality parameters, histopathology, antioxidant status, and hormone levels were tested. The levels of gonadotropin-releasing hormone (GnRH) and gonadotropin-inhibitory hormone in the hypothalamus were detected by western blot.

**Results:**

Compared with the VCL-vehicle group (41.14%), administration of VB significantly increased the sperm viability (59.29, 65.45, 84.93%). VB groups showed higher Johnson’s score (3.57 ± 0.15, 4.71 ± 0.26, 7.93 ± 0.37) than VCL-vehicle group (2.72 ± 0.24). Antioxidant status and hormone levels alterations were also observed. Meanwhile, the mean number of apoptotic tubules (8.15 ± 0.96, 6.61 ± 1.05, 2.17 ± 0.08) and apoptotic index showed a marked decrease. Compared with the VCL-vehicle group (0.21 ± 0.09), the VB groups (0.36 ± 0.07, 0.42 ± 0.06, 0.88 ± 0.10) showed considerable increases in GnRH.

**Discussion and conclusions:**

VB has protective effects on reproductive organs and VB may be therapeutically useful in the treatment of varicocele through up-regulation of the HPG axis.

## Introduction

Varicocele (VCL), which is classified as male-factor infertility, is an abnormal venous dilation of the pampiniform plexus within the scrotum (Agarwal and Esteves 2016; Nevoux et al. 2011; Reed-Maldonado and Madden 2019). VCL is the leading cause of abnormal semen, low sperm count, decreased sperm motility, and abnormal sperm morphology (Alsaikhan et al. 2016; Rotker and Sigman 2016; Kathrins 2017). In recent years, the pathophysiologic mechanisms for improving VCL-induced damage to sperm quality and testicular atrophy have been extensively studied. Previous research established that the mechanisms of VCL-induced testicular dysfunction include increased temperature, inflammatory cytokines, oxidative stress, endoplasmic reticulum stress, hormonal imbalance, and apoptosis (Roque and Esteves 2018; Pagani et al. 2019). However, the exact mechanism has yet to be fully elucidated (Chiba et al. 2016).

Verbascoside (VB), also known as acteoside, was first isolated from mullein (*Verbascum sinuatum* L. [Scrophulariaceae]), and 23 other plant families (Galli et al. 2020). VB is a phenylethanoid consisting of a cinnamic acid and hydroxyphenylethyl moieties attached to a β-glucopyranose through a glycosidic bond. (Alipieva et al. 2014). VB has *in vitro* and *in vivo* biological activities, including antioxidant, anti-inflammatory, antibacterial, antitumor, antifungal, antinociceptive, and immunomodulatory effects as well as free radical scavenging (Zhu et al. 2016; Etemad et al. 2016; Wan et al. 2016). Moreover, VB showed antioxidative and hepatoprotective activities in a CCl_4_-induced liver injury model, and a possible mechanism could be mediated through reducing oxidative stress (Maquiaveli et al. 2017; Attia et al. 2018).

Medicinal plants are used in the treatment of various diseases. The search for alternative approaches or drugs, particularly from medicinal plants (Karna et al. 2019), has become a potential modality of medical treatment for VCL. Recent studies have demonstrated that VB may be specifically involved in the promotion of poor sperm quality and testicular toxicity in rats through up-regulation of steroidogenesis enzymes (Maquiaveli et al. 2016), but there is no clear evidence of its effectiveness in VCL. Therefore, in this study, we established an experimental VCL model to determine the role and mechanism of VB on experimental VCL, as well as to provide a theoretical basis for exploring the molecular mechanisms of experimental VCL.

## Materials and methods

### Animals and drugs

All animals were handled according to the Guide for the Care and Use of Laboratory Animals of the National Institutes of Health (National Research Council [US] Institute for Laboratory Animal Research, 1996), and all experiments were approved by the animal ethics committee of Shandong University of Traditional Chinese Medicine (NO.: SDUTCM20190513012). Sixty male specific-pathogen-free Sprague-Dawley (SD) rats, weighing 300 ± 20 g, were purchased from Jinan Pengyue Experimental Animal Breeding Co., Ltd. (license number SCXK [Lu] 20140007; Jinan, China). The animals were housed in polycarbonate cages in a standard room with a natural 12 h light/dark schedule cycle at standard temperature (21 ± 3 °C) and humidity (45–65%). The animals were fed with a standard diet and water *ad libitum*.

VB with a purity of >99% was purchased from Sigma Aldrich (St. Louis, MO, USA).

### Animal grouping and treatment

After one week of acclimatization, the animals were divided into six groups (*n* = 10): control (healthy rats without induction of VCL), control-sham (simple laparotomy without additional intervention), VCL-vehicle (VCL-induced controls, normal saline, same volume), and three VCL-induced rats treated with VB: VCL + VB50 (VCL-induced rats administered 50 mg/kg VB), VCL + VB100 (VCL-induced rats administered 100 mg/kg VB), and VCL + VB200 (VCL-induced rats administered 200 mg/kg VB).

To induce experimental VCL, rats were anaesthetized via an intraperitoneal injection of ketamine (60 mg/kg) and xylazine (1 mg/kg). An abdominal midline incision of 3–4 mm in length was made. The left renal and internal spermatic veins were located through the incision and cleared of adherent tissue, and the insertion point was detected. A wire (0.85 mm) was placed parallel to the left renal vein and a 4–0 silk suture was used for ligation around the wire and left renal vein proximal to the inferior vena cava (Hassani-Bafrani et al. 2019). The incision was then sutured with 3–0 silk sutures. Administration of VB was started at 30 days after VCL induction and performed daily for 4 weeks.

VB doses administered to rats in this study were selected based on previous reports (Amin et al. 2016). The period of VCL damage induction was chosen according to previous studies (Soni et al. 2018; Karna et al. 2019). The full spermatogenic cycle, including spermatocytogenesis, meiosis, and spermiogenesis, requires 40–45 days in the rat model, and the epididymal transit of spermatozoa takes approximately 1 week in rats (Türk et al. 2016; Dehghani et al. 2019); thus, the treatment period was set at 58 days.

### Sample collection and homogenate preparation

Fifty-eight days after surgery, the animals were weighed and then sacrificed. The hypothalamus was removed, transferred to liquid nitrogen, frozen, and stored at −80 °C. The abdominal cavity was opened, and the testicles were quickly removed and weighed. Half of the tissues were fixed in Bouin’s fixative (Sigma-Aldrich, St. Louis, MO, USA) for histological and apoptotic evaluation. The remaining samples were stored at −80 °C for further assays (Hassani-Bafrani et al. 2019).

### Histopathological examination

Testes were removed from the freezer, separated from the connective tissue, minced, and placed in separate 1.5 mL microcentrifuge tubes. Upon adding pre-warmed (37 °C) normal saline, the tissue was suspended at 37 °C for 5 min. Sperm concentration and viability were evaluated. The seminiferous tubules were rated for their modified spermatogenesis index by Johnson’s score on a scale of 0–10 indicating a range of no cells to complete spermatogenesis (Johnsen 1970).

### Sperm quality analysis

Testes were prepared as described for histopathological examination. Sperm concentration and viability were evaluated using a sperm counting chamber (Sperm Metre; Sperm Processor, Aurangabad, India) and light microscopy, respectively (Türk et al. 2016).

### Determination of serum LH, FSH, testosterone, and GnRH

The levels of testosterone (Roche Diagnostics, Mannheim, Germany), luteinizing hormone (LH; RPN 2562, Amersham, UK), follicle-stimulating hormone (FSH; RPN 2560, Amersham, UK), and gonadotropin-releasing hormone (GnRH; Cusabio Technology, LLC, Wuhan, China) in serum were measured by enzyme-linked immunosorbent assay (Gan and Patel 2013) according to the manufacturer’s instructions (R&D Systems, Minneapolis, MN, USA).

### Biochemical analyses for evaluating testicular SOD, GSH, GPx, and MDA levels

The activity of superoxide dismutase (SOD) in testicular tissue was measured by the xanthine oxidase assay (Paoletti and Mocali 1990). Glutathione (GSH) activity was measured by a colorimetric method. The thiobarbituric acid assay was performed to detect malondialdehyde (MDA). Glutathione peroxidase (GPx) activity was measured using commercial kits. All procedures were conducted according to manufacturer’s instructions (R&D Systems). SOD and GPx activities were expressed as units (U) of enzyme activity (U/mg protein), and MDA and GSH (Sohrabipour et al. 2013) were expressed as μmol/mg of protein.

### Measurement of germ cell apoptosis by the TUNEL assay

To quantitatively assess apoptosis, slides with testicular tissue were stained according to the TUNEL immunohistochemical assay method (Migone et al. 2017). Apoptotic nuclei in specimens were assessed with an *in situ* apoptosis detection kit (Trevigen, Gaithersburg, MD, USA) according to the manufacturer’s protocol. Finally, the number of seminiferous tubules with apoptotic cell signals was counted per cross-section at 200× magnification. At least 30 seminiferous tubules from each slide were counted to determine the number of apoptotic cells. The mean number of TUNEL-positive cells per tubule cross-section and the ratio of TUNEL-positive tubules to the total number of tubules were also obtained.

### Western blot

Protein lysates were used to extract the proteins in the hypothalamus tissues. Protein concentrations were determined by the bicinchoninic acid method (Jin et al. 2018), and SDS-PAGE was performed to resolve the proteins, which were transferred to polyvinylidene fluoride membranes (Merck, Darmstadt, Germany) at 80 V for 30 min and blocked for 1 h with 5% non-fat dry milk in TBST. Antibodies of GnRH (1:5000, Santa Cruz, CA, USA), gonadotropin-inhibitory hormone (GnIH; 1:8000, Kazuyushi Tsutsui, Tokyo, Japan) were diluted in 3% bovine serum albumin in TBST and incubated with horseradish peroxidase goat anti-rabbit IgG (1:2000, Proteintech, USA) for 1 h. An electrochemiluminescence system was used to detect the signal. The protein expression level was standardized to that of β-actin, and greyscale scanning and quantification were performed using Image J (NIH, Bethesda, MD, USA).

### Statistical method

SPSS 20.0 (SPSS IBM, Armonk, NY, USA) was used to analyze the monitoring data. Data were expressed as mean ± standard deviation (mean ± SD). Student’s *t*-test was used for data analysis between two groups. Comparisons among multiple groups were performed using one-way analysis of variance with Tukey’s *post hoc* multiple comparison test, and *p* < 0.05 was considered statistically significant.

## Results

### VB ameliorated the reduction in the left testis weight

To determine the effect of VB in VCL rats, we weighed the body and reproductive organs. As shown in Table 1, there were no differences between the groups in terms of body weight and right testis weight (*p* > 0.05; Table 1). Concomitantly, compared with the control group, the VCL-vehicle group showed a marked reduction in left testis weight (*p* < 0.05; Table 1). Compared with the VCL-vehicle group, the VB groups revealed a considerable increase in left testis weight (*p* < 0.05; Table 1). No difference occurred between the control-sham and control groups (*p* > 0.05; Table 1). Treatment with VB (50, 100, and 200 mg/kg) dose-dependently protected the left testis from reductions in weight. Moreover, the left testis weight significantly increased in the group treated with 200 mg/kg VB.

**Table 1.** The effect of the VB on weights of body and testis.

	Control	Control-sham	VCL-vehicle	VCL + VB50	VCL + VB 100	VCL + VB 200
Initial body weight (g)	349.73 ± 16.52	351.23 ± 11.72	346.29 ± 18.73	348.93 ± 16.78	349.38 ± 18.25	353.26 ± 24.12
Final boday weight (g)	439.23 ± 25.78	413.56 ± 28.35	403.23 ± 29.43	408.15 ± 36.72	402.13 ± 14.31	407.25 ± 36.13
Right testis (g)	2.35 ± 0.21	2.13 ± 0.14	2.07 ± 0.09	2.06 ± 0.15	1.99 ± 0.17	2.01 ± 0.05
Left testis (g)	2.36 ± 0.13	2.10 ± 0.16	1.14 ± 0.26^#^	1.29 ± 0.31^#^	1.79 ± 0.21*	1.93 ± 0.16*

Data are presented in mean ± SD. *p < 0.05 vs. VCL-vehicle group, ^#^*p* < 0.05 vs. Control group.

### VB improved sperm quality parameters

To verify the effects of VB on sperm quality parameters, we first tested whether VB could increase the sperm count and viability. Results showed that the sperm count and viability were reduced in the VCL-vehicle group (*p* < 0.05; Table 2); compared with that group, the VB groups showed a marked increase in sperm concentration and viability (*p* < 0.05; Table 2). No difference was observed between the control-sham and control groups (*p* > 0.05; Table 2). Treatment with VB (50, 100 and 200 mg/kg) increased the sperm count and viability in a dose-dependent manner. Moreover, the sperm concentration and viability significantly increased in the group treated with 200 mg/kg VB.

**Table 2.** The effect of the VB on sperm count and viability.

	Control	Control-sham	VCL-vehicle	VCL + VB 50	VCL + VB 100	VCL + VB 200
Sperm count (×10^6^/mL)	129.36 ± 9.13	114.70 ± 8.61	45.16 ± 12.86^#^	69.23 ± 10.29*	71.69 ± 8.41*	96.93 ± 9.16*
Sperm viability (%)	80.23 ± 0.13	81.70 ± 7.16	41.14 ± 9.92^#^	59.29 ± 6.27*	65.45 ± 5.32*	84.93 ± 4.16*

Data are presented in mean ± SD. *p < 0.05 vs. VCL-vehicle group, ^#^*p* < 0.05 vs. Control group.

### VB regulated histological changes in testes

**Figure 1.**
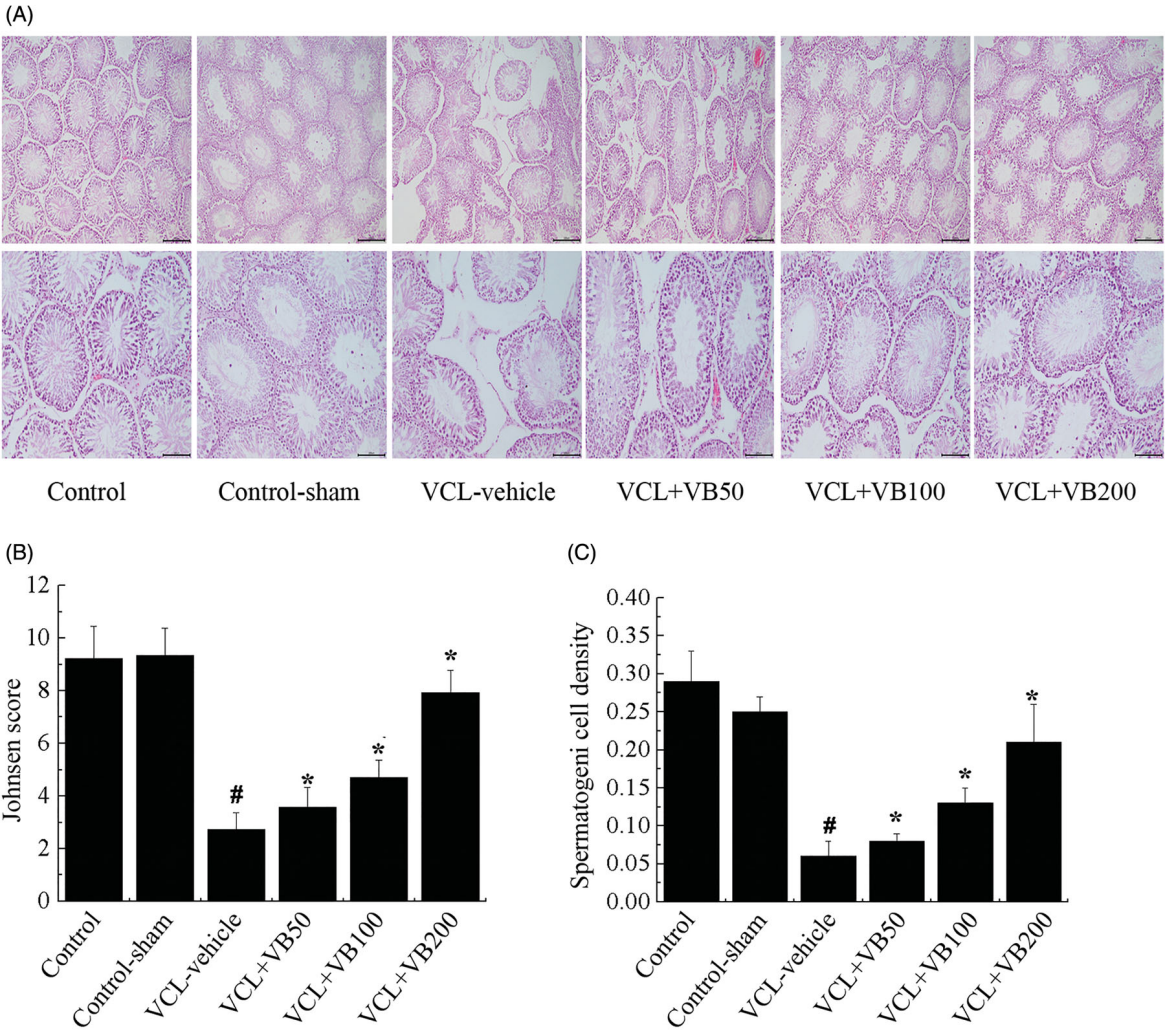
Histologic findings in testis. (A) Histological changes in the testis. Original magnifications 100×, 200×. (B) Spermatogenic cell density. (C) Johnsen’s score of the seminiferous tubules. *p < 0.05 vs. VCL-vehicle group, ^#^*p* < 0.05 vs. Control group.

To verify the effects of VB on testicular tissues, we conducted haematoxylin & eosin staining. As shown in Figure 1, testes in the control and control-sham groups had normal morphologic characteristics. The seminiferous tubules were closely packed, the spermatogenic epithelium exhibited normal morphology, and no vacuolization of the germinal epithelium was observed.

In the VCL-vehicle group, the seminiferous tubules were atrophied and appeared irregular with a loss of normal morphology. What’s more, with the decrease in spermatogenic cells, the atrophic cells and intercellular vacuoles caused widening of the seminiferous lumen. Compared with the control group, there was a thinner spermatogenic epithelium, and vacuolization of the germinal epithelium was observed (Figure 1). Additionally, Johnson’s score was significantly decreased (*p* < 0.05; Figure 1).

Compared with the VCL-vehicle group, all VB groups showed normal developed histopathological features and an increased Johnson’s score (*p* < 0.05; Figure 1). As for the VCL + VB50 (3.57 ± 0.15) and VCL + VB100 (4.71 ± 0.26) groups, the rate of injury induced by VCL was reduced in comparison with that of the VCL-vehicle group (2.72 ± 0.24). Nevertheless, various cell types were also visible in tubules, and the number of germ cells decreased. Treatment with VB (50, 100, and 200 mg/kg) dose-dependently protected the left testis from damage. The group treated with 200 mg/kg VB (7.93 ± 0.37) showed significant histological improvement with a mild degree of necrosis comparable to that of the control group.

### VB decreased apoptosis

**Figure 2.**
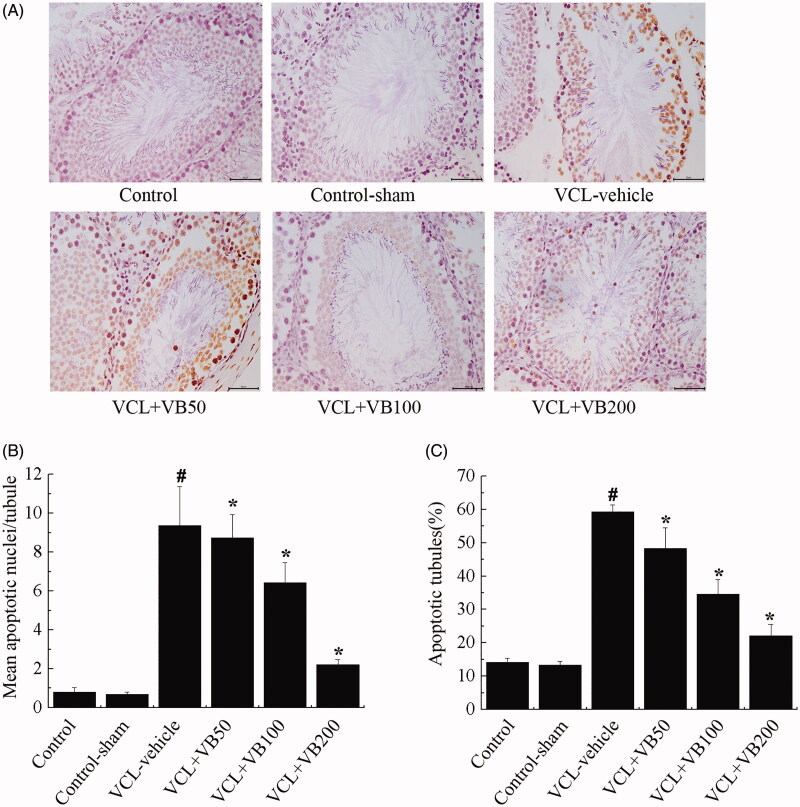
TUNEL assays for the measurement of germ cell apoptosis. (A) TUNEL-staining in each group. Original magnifications 400× (B) Mean apoptotic nuclei/tubule. (C) Apoptotic tubules (%). *p < 0.05 vs. VCL-vehicle group, ^#^*p* < 0.05 vs. Control group.

To elucidate the effects of VB on apoptosis in testes, the mean number of apoptotic tubules and apoptotic index were examined. Following VCL induction, the mean number of apoptotic tubules and apoptotic index were significantly increased. Concomitantly, compared with the control group, these values were markedly increased in the VCL-vehicle group (*p* < 0.05; Figure 2). Compared with the VCL-vehicle group (9.89 ± 1.13), the VB groups (8.15 ± 0.96, 6.61 ± 1.05, 2.17 ± 0.08) showed a marked decrease in the mean number of apoptotic tubules (*p* < 0.05; Figure 2). Significantly lower apoptotic indices were also seen in VB groups (48.31 ± 6.20, 34.64 ± 4.35, 22.05 ± 3.48) compared with the VCL-vehicle group (59.32 ± 2.03). Mean apoptotic tubule numbers and apoptotic index levels did not differ between the control-sham and control groups (*p >* 0.05; Figure 2). Treatment with VB (50, 100 and 200 mg/kg) resulted in a dose-dependent decrease in apoptosis. Furthermore, the mean number of apoptotic tubules and apoptotic index significantly decreased in the group treated with 200 mg/kg VB.

### VB regulated the hormone levels

**Figure 3.**
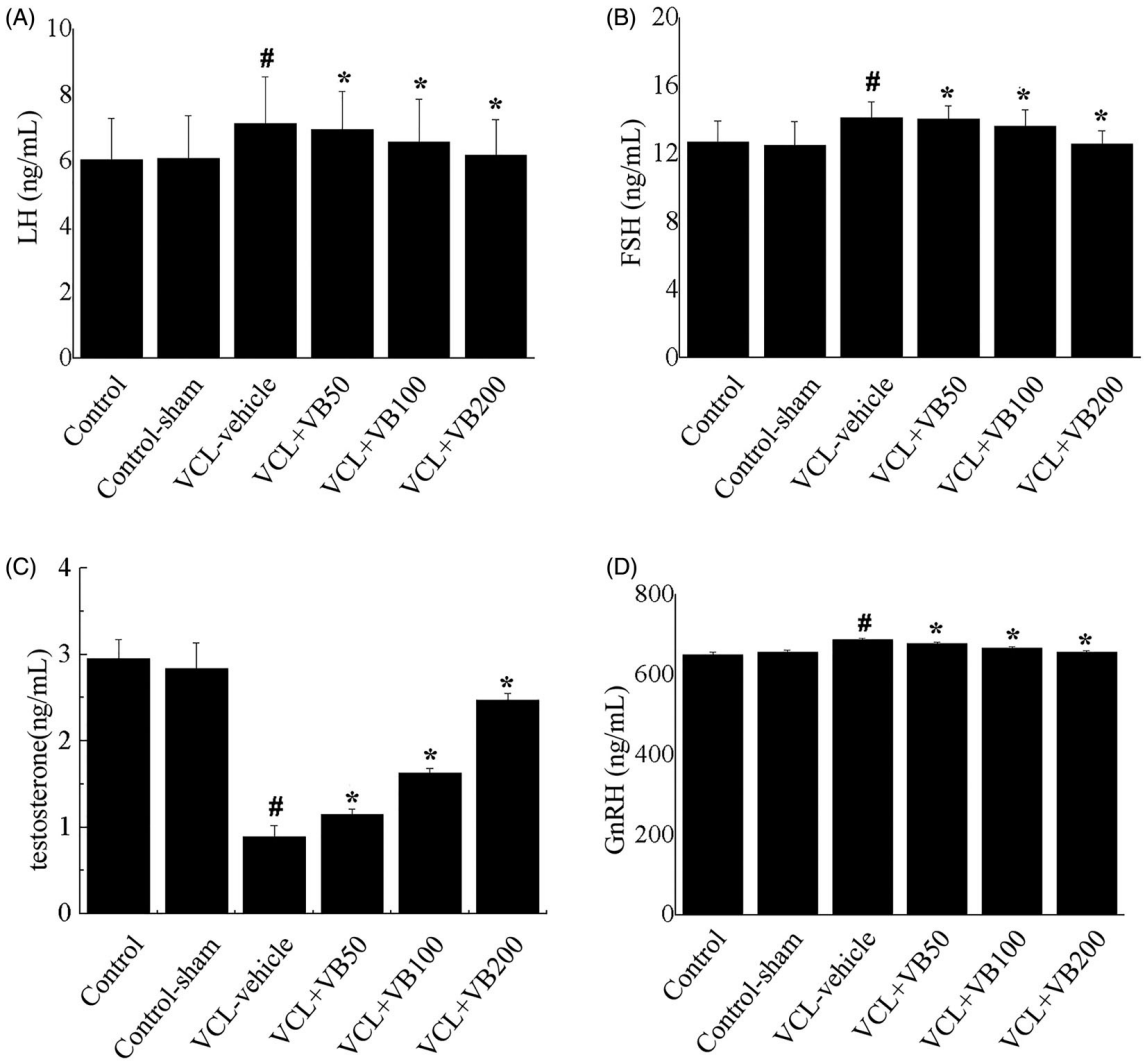
The hormone levels in each group. (A) LH levels. (B) FSH levels. (C) testosterone levels. (D) GnRH levels. *p < 0.05 vs. VCL-vehicle group, ^#^*p* < 0.05 vs. Control group.

To elucidate the effects of VB on hormone levels, we examined the levels of testosterone, LH, FH, and GnRH. After VCL induction, testosterone was decreased, while LH, FH, and GnRH were increased. Compared with the control group, the VCL-vehicle group showed a marked reduction in testosterone levels (*p* < 0.05; Figure 3(A)), while LH, FH, and GnRH were significantly increased (*p* < 0.05, Figure 3(B–D)). Concomitantly, compared with the VCL-vehicle group, all VB groups showed a marked increase in testosterone levels (*p* < 0.05; Figure 3(A)), while LH, FH, and GnRH levels exhibited significant reductions (*p* < 0.05, Figure 3(B–D)). There was no difference between the control-sham and control groups (*p* > 0.05; Figure 3). After treatment with 50, 100, and 200 mg/kg VB, the LH, FH, and GnRH levels were significantly decreased while testosterone levels were increased. The testosterone, LH, FH, and GnRH levels in the group treated with VB at 200 mg/kg were close to those of the control group.

### VB regulated testicular antioxidant status

**Figure 4.**
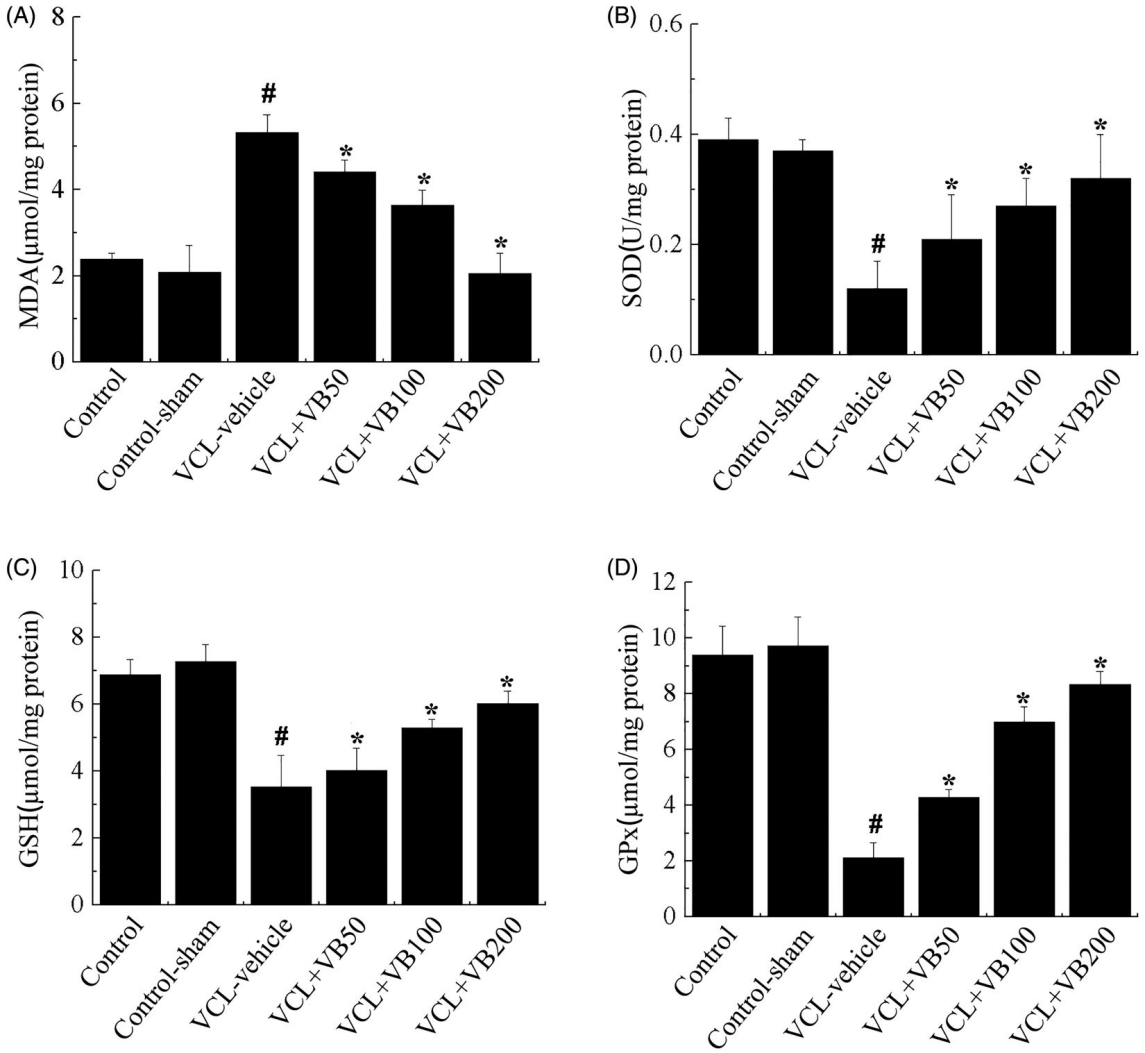
Testicular antioxidant status in each group. (A) MDA levels. (B) SOD levels. (C) GSH levels. (D) GPx levels. *p < 0.05 vs. VCL-vehicle group, ^#^*p* < 0.05 vs. Control group.

To evaluate the effects of VB on antioxidant enzymes, we next examined how the expression of SOD, GSH, GPx, and MDA changed. SOD, GSH, and GPs levels were significantly decreased after VCL induction, while MDA was significantly up-regulated (*p* < 0.05, Figure 4(A)). Concomitantly, compared with the VCL-vehicle group, the VB groups showed marked increases in SOD, GSH, and GPx levels (*p* < 0.05; Figure 4(B–D)), while MDA levels were significantly reduced (*p* < 0.05, Figure 4). There was no difference between the control-sham and control groups (*p* > 0.05; Figure 4). Treatment with VB (50, 100, and 200 mg/kg) resulted in the up-regulation of SOD, GSH, and GPx levels and a reduction in MDA levels (*p* < 0.05). SOD, GSH, GPx, and MDA levels in the group treated with 200 mg/kg VB were close to those of the control group.

### VB regulated the protein levels of GnRH and GnIH

**Figure 5.**
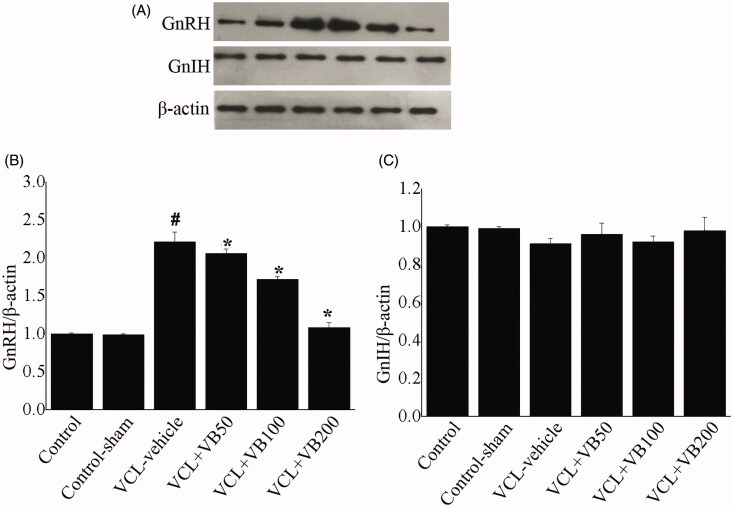
The protein levels of GnRH and GnIH. (A) Western-blot was performed to determine the GnRH and GnIH level in each group. (B) Quantification of GnRH expression. (C) Quantification of GnIH expression. *p < 0.05 vs. VCL-vehicle group, ^#^*p* < 0.05 vs. Control group.

To understand the mechanism by which VB regulates VCL-induced damage, we examined the expression of GnRH and GnIH (Figure 5). Proteins levels were compared using the ratios of GnRH and GnIH levels to β-actin levels to evaluate the activation of proteins in hypothalamus tissues.

Compared with the control group, the VCL-vehicle group showed a markedly increased GnRH level (*p* < 0.05; Figure 5). Compared with the VCL-vehicle group (0.21 ± 0.09), the VB groups (0.36 ± 0.07, 0.42 ± 0.06, 0.88 ± 0.10) showed considerable increases in GnRH (*p* < 0.05; Figure 5). There was no difference between the control-sham and control groups (*p* > 0.05; Figure 5).

As for the GnIH level, there were no significant differences between the groups (*p* > 0.05; Figure 5).

## Discussion

VCL is characterized by high morbidity, and the incidence of this disease continues to rise due to the environment, heredity, lifestyle, and other effects (Agarwal and Esteves 2016; Attia et al. 2018). The mechanism of VCL and spermatogenesis is a multistep process that involves numerous biological signals and stimuli. Although new technologies have developed rapidly in recent years, the pathophysiology of VCL-related infertility remains unclear (Nevoux et al. 2011; Roque and Esteves 2018). Spermatogenesis and the regulatory role of the HPG axis on functional integration of the reproductive system are the most frequent causes of high morbidity (De Martino et al. 2003). The present study demonstrated the ability of VB to protect against VCL-induced testicular damage and dysregulation of the HPG axis in a VCL rat model.

There were no changes in body weight and right testis weight, and VB treatment in VCL rats did not lead to a decrease in body weight as a consequence of a loss of appetite. However, the left testis weights in the VCL-induced group were significantly reduced, whereas the VB groups showed a marked increase in the left testis weight. VB was shown to repress the VCL-induced weight loss in the left testis. Previous studies have shown that the sperm concentration and viability were decreased in a VCL rat model (Kathrins 2018). Consistent with that study, we observed a prominent increase in sperm quality in the 200 mg/kg VB group.

However, the results also showed that many histopathologic changes occurred in VCL-induced rats, such as a decreased tubular diameter, spermatogenic arrest, and decreased number of germ cells. To elucidate this situation, the histological findings showed that administration of 200 mg/kg VB could attenuate the degenerative alterations in VCL-induced rats.

Recent studies have shown that testicular tissue has highly unsaturated fatty acids (Cho et al. 2019). Accordingly, it is vulnerable to oxidative stress (Pastuszak and Wang 2015). Based on our records, VCL was associated with reduced MDA accumulation, reduced GSH content, and diminished SOD and GPx activity, which leads to oxidative damage of the testicular physiology, hindered sperm function, and even DNA damage in the testicular tissue of rats. Treatment with VB significantly reduced the number of apoptotic cells, decreased the MDA level, and increased the activity of antioxidant enzymes in the VCL-induced rats. These results were consistent with previous reports.

According to several studies, FSH and LH regulate testosterone secretion by Leydig cells (Sand et al. 2013; Fischer et al. 2019). They are required for the growth and maintenance of the seminiferous tubules and spermatogenesis. LH is a very important hormone responsible for spermatogenesis. As for LH and testosterone, LH can stimulate testosterone production by Leydig cells. Testosterone secretion is positively correlated with the modification of LH secretion (Ivell et al. 2014; Burow et al. 2019; Mohammadi et al. 2019). Thus, we confirmed that regulatory effect of 200 mg/kg VB on FSH, LH, and testosterone.

It is known that the HPG axis is a central system that has complex relationships with other systems mediating the acquisition and maintenance of reproductive ability. GnIH neurons interact directly with GnRH neurons mediated by a G-protein coupled receptor (Peper et al. 2010). VCL disturbs hormone levels via its effects on the HPG axis by decreasing testosterone levels while increasing GnRH, FSH, and LH levels in serum. Previous research indicated that VCL increases the levels of GnRH, FSH, and LH (Blevrakis et al. 2016; Zhang et al. 2016). In our study, VB was determined to regulate the protein levels of GnRH, FSH, LH, and testosterone. There are numerous exogenous and endogenous factors that can induce excessive ROS production, thus causing oxidative stress. What’s more, oxidative stress negatively affects male reproductive functions and may induce infertility by affecting the HPG axis. These results confirmed the findings of previous studies.

## Conclusions

The results revealed that VB could prevent the experimental VCL-induced reduction in sperm count and quality, hormonal disturbances, testicular oxidative stress, testicular atrophy and histopathological lesions in rats through regulation of the HPG axis. Our results suggest that VB is a promising agent for the treatment of experimental VCL and could serve as a candidate for VCL therapy.
